# Impact of the adjuvant management and risk factors on survival in FIGO stage 3 endometrial cancer patients

**DOI:** 10.3389/fonc.2023.1035511

**Published:** 2023-04-06

**Authors:** Nora Tong, Aalok Kumar, Gerald Gelowitz, Anna Tinker, Caroline Holloway, Jenny Ko

**Affiliations:** ^1^ Faculty of Medicine, University of British Columbia, Vancouver, BC, Canada; ^2^ Department of Medical Oncology, British Columbia (BC) Cancer - Surrey, Surrey, BC, Canada; ^3^ Department of Radiation Oncology, British Columbia (BC) Cancer – Abbotsford, Abbotsford, BC, Canada; ^4^ Department of Medical Oncology, British Columbia (BC) Cancer - Vancouver, Vancouver, BC, Canada; ^5^ Department of Radiation Oncology, British Columbia (BC) Cancer - Victoria, Victoria, BC, Canada; ^6^ Department of Medical Oncology, British Columbia (BC) Cancer – Abbotsford, Abbotsford, BC, Canada

**Keywords:** gynecology, radiotherapy, chemotherapy, uterine cancer, endometrial neoplasms, uterine neoplasms

## Abstract

**Objective:**

Patients with FIGO stage III endometrial cancer routinely receive adjuvant therapy. The purpose of this study was to evaluate overall survival (OS) and disease-free survival (DFS) in patients with stage IIIA to IIIC2 patients by treatment modality received and risk factors.

**Materials/methods:**

Patients with stage III endometrial cancer treated from 2000-2010 were identified in the provincial cancer registry. Clinicopathologic characteristics, adjuvant treatments and outcomes were compared using descriptive and multivariable analyses.

**Results:**

261 patients had stage 3 endometrial cancer, 132 with stage IIIA, 9 with IIIB, 85 with IIIC1 and 35 with IIIC2. 39 had FIGO grade 1 disease; 73, grade 2; 147, grade 3. 160 had endometrioid and 35 had serous carcinoma. 161 patients received sequential adjuvant chemotherapy (CT) and radiotherapy (RT); 33 received RT only; 32 received CT only; 35 received neither. 5-year (5Y) DFS and OS were similar among stage IIIA (DFS 46.7%, OS 58.5%), IIIB (DFS 50.8%, OS 58.5%), IIIC1 (DFS 44%, OS 49.9%) and IIIC2 (DFS 42%, OS 41.6%). Use of adjuvant RT was associated with improved median DFS (53.7 vs 14.7m, p<0.00001) and OS (61.9 vs 25.7m, p<0.00001) compared to no RT. Likewise, use of adjuvant CT was also associated with improved DFS (54.8 vs 16.5m, p<0.00001) and OS (62.9 vs 26.5m, p<0.00001) compared to no CT. Those who received both chemotherapy and radiotherapy had better outcomes with 5-year DFS (58.3%) and OS (65.2%), compared with those who received monotherapy. On multivariate analysis, grade 3 disease, deep myometrial invasion >50%, and no adjuvant RT or CT were identified as adversely impacting DFS and OS.

**Conclusion:**

In stage III endometrial cancer patients, use of both chemotherapy and radiation therapy was associated with improved DFS and OS and therefore should be recommended in all eligible patients after resection.

## 1 Introduction

Endometrial cancer is the sixth most common cancer in females worldwide, with 417,000 new cases and 97,000 deaths in 2020 (1). Incidence rates have increased in many countries since the late 1990s (1). Women with stage III and IV endometrial cancer have a worse prognosis with a 5-year survival of 57-66% and 20-26%, respectively (2).

Patients with stage III endometrial cancer are typically managed with surgery and adjuvant therapy (3). In the most recent guideline by the European Society of Gynaecological Oncology (ESGO), it is recommended that treatment for clinically and molecularly high-risk endometrial carcinomas include external beam radiation and adjuvant chemotherapy (3). There continues to be areas of uncertainty despite the guideline. Although stage III endometrial cancer is generally considered to be high-risk for recurrence, the recommendations for adjuvant treatment become unclear in each molecular subtype. It is also unclear whether the adjuvant treatments should be delivered sequentially or concurrently, as sequential chemotherapy and radiation therapy after surgery, a common treatment regimen especially for stage III endometrial cancer, was not examined in phase III trials (4).

The purpose of our study was to evaluate overall survival (OS) and disease-free survival (DFS) following sequential chemotherapy and radiation therapy in patients with FIGO stage IIIA to IIIC2 endometrial cancer over a ten-year period.

## 2 Materials and methods

### 2.1 Study design and conduct

This was a multi-center retrospective cohort study within six provincial centers in British Columbia, Canada. The study was approved by the BC Cancer Agency Research Ethics Board and conducted according to the ICH Harmonized Tripartite Guidelines for Good Clinical Practice, Declaration of Helsinki, and the National Statement on Ethical Conduct in Research Involving Humans. We included in the study consecutive patients with stage III endometrial cancer, confirmed by the authors through chart review based on the 2009 FIGO staging system (5), diagnosed between Jan 1, 2000, to Dec 31, 2010, identified in the BC Cancer registry. Investigators performed a chart review and collected demographic data, covariates of prognostic significance, types of chemotherapy, radiation therapy and drugs used, frequency of imaging, and outcomes such as DFS and OS.

We summarized clinicopathologic characteristics including assessment of histology, myometrial invasion, lymphovascular invasion, locally involved extrauterine organs, and size of endometrial primary. Treatment characteristics compared included type of surgery, use of adjuvant chemotherapy and adjuvant radiotherapy, external beam radiation technique, fields, and dose.

OS was defined as from the date the patient received their diagnosis until the date they died or were last followed-up. Patient identifying information was concealed at the last follow-up. DFS was from the date of diagnosis until the date the patient relapsed, progressed, died, or were last followed up. Patient identifying information was concealed at the last follow-up visit.

### 2.2 Eligibility

All consecutive patients diagnosed with stage III endometrial cancer between Jan 1, 2000, to Dec 31, 2010 were included in the study, then reviewed for eligibility. Endometrial cancer was staged according to the 2009 International Federation of Gynecology and Obstetrics (FIGO) staging by reviewing all pathologic and radiologic studies for individual patients; specifically, patients who were previously assigned stage IIIA disease by pelvic washing cytology only according to the 1998 FIGO staging were removed from the analysis (5). Patients were excluded if they were lost to follow up, incorrectly staged, had incomplete chart data regarding pathology and treatment, if they moved to another province, or if there was no death date listed. 108 patients were excluded due to incorrect staging or a lack of meaningful data. None of the participants were lost to follow up.

### 2.3 Statistical analyses

OS and DFS were assessed using Kaplan-Meier survival analyses. The separation of survival between treatments was assessed with log-rank statistics. Multivariate cox proportional hazard regression models were used to assess variables that could have influenced survival outcomes. The categorical variables assessed were stage, grade, presence or absence of myometrial invasion, radiation, and chemotherapy. A p-value less than 0.05 was considered statistically significant.

## 3 Results

We collected data from a total of 261 patients from the six provincial cancer centers in British Columbia, Canada (**Table 1**). Of those patients, 132 had stage 3A, 9 had 3B, 85 had 3C1, and 35 had 3C2 endometrial cancer. 39 were classified with FIGO grade 1 disease, 73 with grade 2, and 147 with grade 3. 160 patients had endometrioid and 35 had serous carcinoma. 170 patients had >50% myometrial invasion and 162 had presence of lymphovascular invasion. In terms of surgery, total abdominal hysterectomy was done in all but 3 patients who were poor operative candidates. 179 (68.6%) had pelvic washing and 78 (29.9%) had omentectomy. 127 (48.7%) had at least 1 pelvic lymph node resected, and 17 (6.5%) had at least 1 para-aortic lymph node resected. Lymph node assessment was otherwise performed with either CT or MR imaging. After surgery, patients received sequential adjuvant chemotherapy and radiotherapy (n=159), radiotherapy only (n=32), chemotherapy only (n=32), or neither (n=35) (**Table 1**). Of the 191 patients who received adjuvant chemotherapy, 95.8% received carboplatin/paclitaxel. Of the 191 patients who received adjuvant radiation therapy, 27% received external beam radiation, 1% received brachytherapy alone, and 71.9% received both.

**Table 1:** Patient and disease characteristics.

Characteristic	Type	Number of patients	Percentage
*Stage*	3A	132	50.6%
	3B	9	3.4%
	3C1	85	32.6%
	3C2	35	13.4%
*FIGO grade*	1	39	14.9%
	2	73	28.0%
	3	147	56.3%
	Unknown	2	0.8%
*Histology*	Endometrioid - Grade 1: 36 (22.5%) - Grade 2: 62 (38.8%) - Grade 3: 62 (38.8%)	160	61.3%
	Serous carcinoma	35	13.4%
	Clear cell	10	3.8%
	Mucinous	1	0.4%
	Undifferentiated/carcinosarcoma/MMMT	5	1.9%
	Mixed	48	18.4%
	Unknown	2	0.8%
*Myometrial invasion*	>50%	170	65.1%
*Lymphovascular invasion*	Present	162	62.0%
*Adjuvant therapy*	Received both chemotherapy and radiation therapy	159	60.9%
	Received chemotherapy only	32	12.3%
	Received radiotherapy only	33	12.6%
	Received neither	35	13.4%
	Unknown	2	0.8%
*Adjuvant radiation therapy (n=192)*	Both external beam radiation therapy (EBRT) and brachytherapy	138	71.9%
	EBRT only • 4-field technique = 178 • Intensity-modulated radiation therapy = 12 • Other = 2	52	27.1%
	Brachytherapy only	2	1.0%
*Adjuvant systemic therapy (n=191)*	Carboplatin/paclitaxel	183	95.8%
	Carboplatin/docetaxel	2	1.0%
	Single agent platinum	5	2.6%
	Non-platinum agent (clinical trial)	1	0.5%
	Median number of cycles: 3 (range 1-6)		
	Documented grade 3-4 side effects from chemotherapy: 12 (3 febrile neutropenia)		

5-year DFS and OS were similar among patients with stage IIIA (DFS 46.7%, OS 58.5%), IIIB (DFS 50.8%, OS 58.5%), IIIC1 (DFS 44%, OS 49.9%), and IIIC2 (DFS 42%, OS 41.6%) endometrial cancer (**Supplementary Figures 1**, **2**). 10-year DFS and OS were also similar among all sub-stages (IIIA: DFS 27.3%, OS 29.7%; IIIC2: DFS 42%, OS 31.2%).

**Figure 1:**
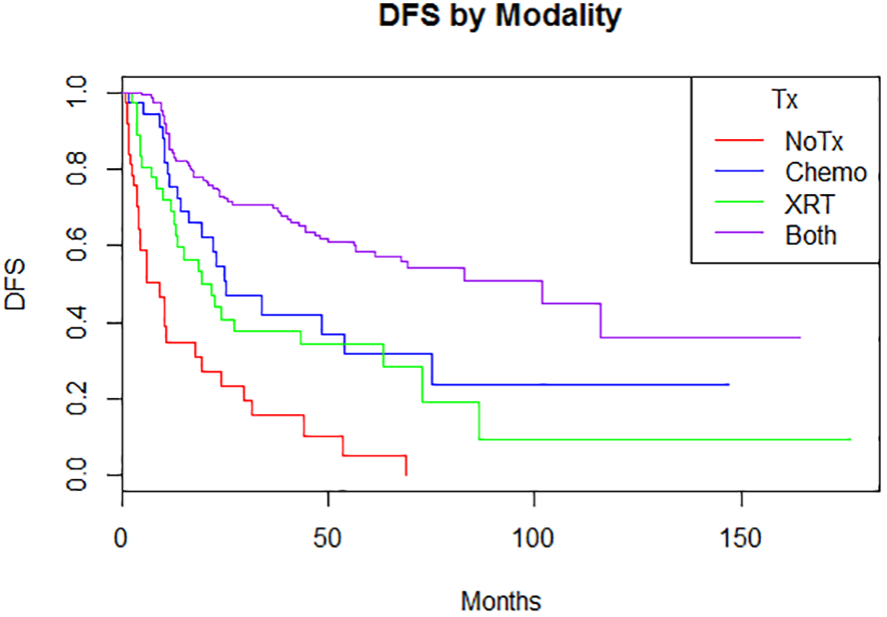
Disease-free survival by modality (p = <0.00001)5-year: no treatment: 5% (0.08-32.1); chemotherapy 31.6% (17.4-57.4); radiation therapy 34.1% (21.5-55.2); both 58.3% (50.4-67.3) 10-year: no treatment: N/A; chemotherapy 23.7% (10.4-53.9); radiation therapy 9.5% (1.7-52.4); both 36.8% (21.0-61.8).

**Figure 2:**
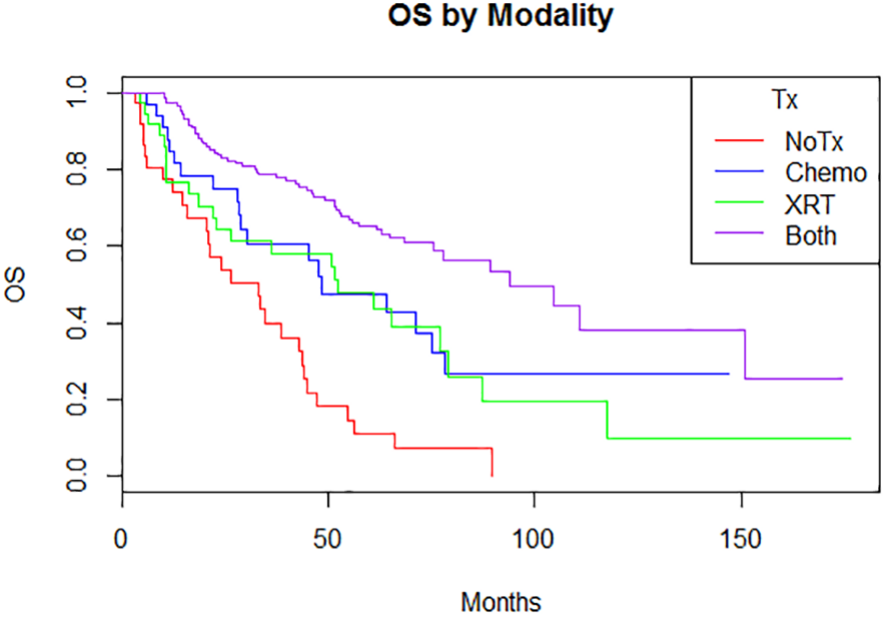
Overall survival by modality (p < 0.00001) 5-year: no treatment: 11% (3.8-31.3); chemotherapy 47.5% (31.9-70.9); radiation therapy 47.8% (33.2-68.8); both 65.2% (57.5-74) 10-year: no treatment: N/A; chemotherapy 26.7% (13.3-53.9); radiation therapy 9.7% (1.8-51.5); both 38.1% (24.4-59.6).

Adjuvant radiotherapy only, chemotherapy only, and sequential radiotherapy and chemotherapy cohorts had longer DFS and OS compared to no adjuvant therapy (**Figures 1**, **2**). Adjuvant radiotherapy was associated with improvements in median DFS (53.7 months vs. 14.7 months, p<0.0001) and OS (61.9 months vs. 25.7 months, p<0001) compared to no radiation therapy. Adjuvant chemotherapy similarly showed improvements in median DFS (54.8 vs. 16.5 months, p<0.00001) and OS (62.9 months vs. 26.5 months, p<0.00001) compared to no chemotherapy. Those who received sequential chemotherapy and radiotherapy had even better outcomes with 5-year DFS (58.3%) and OS (65.2%), compared with those who received radiotherapy only (DFS 34.1%, OS 47.8%) or chemotherapy only (DFS 31.6%, OS 47.5%). None received concurrent chemoradiation, as this option was not offered to patients per BC provincial guidelines.

In multivariable analysis (**Table 2**), older age, grade 3 disease, deep myometrial invasion >50%, and no adjuvant radiotherapy or chemotherapy were associated with shorter DFS and OS. Although non-endometrioid histology had poorer OS (39.8 vs. 77.6 months, p<0.0001) and DFS (22.9 vs. 116.1 months, p<0.0001) than endometrioid histology in univariable analysis, it was not an independent factor for shorter survival in the multivariable analysis (**Table 2**).

**Table 2:** Multivariate analysis of age, stage, grade, myometrial invasion, and adjuvant radiotherapy or chemotherapy on DFS and OS.

	DFS		OS	
	HR	p-value	HR	p-value
*Age*	1.02(1.00-1.04)	0.05	1.03(1.01-1.05)	0.01
*Stage (vs. 3a)* *3b* *3c1* *3c2*	1.66(0.65-4.25) 1.02(0.65-1.59) 1.08(0.61-1.91)	0.29 0.94 0.80	1.17(0.46-1.98) 0.83(0.55-1.26) 1.26(0.74-2.16)	0.74 0.39 0.40
*Grade(vs Grade 1)* *Grade 2* *Grade 3*	1.10(0.49-2.48) 3.20(1.54-6.66)	0.82 0.002	1.42(0.64-3.15) 5.23(2.48-11.03)	0.39 <0.00001
*Histology (vs endometrioid)* *Non-endometrioid*	1.20(0.78-1.84)	0.41	0.93(0.62-1.39)	0.73
*MyoInvas*	2.14(1.37-3.34)	0.0008	2.47(1.52-4)	0.0003
*Radiation*	0.48(0.29-0.74)	0.001	0.39(0.25-0.61)	<0.0001
*Chemo*	0.53(0.34-0.83)	0.005	0.62(0.41-0.96)	0.03

In patients who received radiation therapy, most recurrences were at distant sites (11%) (**Table 3**). Abdominal/pelvic relapses (11%) were also more common than vaginal/pelvic relapse (7%) in patients who received radiation therapy. However, patients who did not receive adjuvant radiation therapy were more likely to have abdominal/pelvic relapse (39%). Distant or visceral metastases were similar between those who received radiation (11%) compared to those who did not (12%).

**Table 3:** Pattern of relapse.

Adjuvant RT	Number of patients	In field vaginal/pelvic relapse	Out of field vaginal/pelvic relapse	Abdominal/pelvic relapse (in and out of fields)	Distant sites or with visceral metastases	Abdominal/pelvic relapse (surgical site with no RT)	More than 1
*Yes*	209	11 (5%)	5 (2%)	23 (11%)	23 (11%)	N/A	18 (9%)
*No*	75	N/A*	N/A	N/A	9 (12%)	29 (39%)	9 (12%)

N/A*: not applicable.

## 4 Discussion

There are areas of uncertainty in defining optimal adjuvant therapy for patients with stage III endometrial cancer (6). Although previous studies have reported on improved outcomes with adjuvant chemotherapy and radiation therapy, what sequence or combination of chemotherapy and radiation therapy is optimal and whether sequential treatment and combined treatment offer similar benefits is largely unknown (3, 7–11). The PORTEC-3 trial was a large prospective trial that showed a survival benefit with chemotherapy (2 cycles of cisplastin) and concurrent radiotherapy, followed by 4 cycles of adjuvant chemotherapy with carboplatin/paclitaxel. The study did not examine sequential chemotherapy and radiotherapy, a common treatment regimen for stage III endometrial cancer (4). Therefore, our study helps fill the gap in understanding the impact of sequential chemoradiation therapy after surgery on outcomes in this population.

Our retrospective study provides a longer, 10-year post-treatment analysis of outcomes for patients with stage III endometrial cancer who have received sequential adjuvant chemotherapy and radiation therapy, chemotherapy only, radiation therapy only, or neither. Our real-world data suggests that patients who received sequential adjuvant chemotherapy and radiation therapy are associated with the best outcomes, even after accounting for other possible prognostic clinical factors. Other retrospective studies report on outcomes of adjuvant radiation therapy with conflicting results. In a study of patients with stage III endometrial cancer, no difference was observed in survival outcomes or disease recurrence in patients who received radiotherapy alone or sequential chemotherapy and radiation therapy (5). A limitation of their study is that patients treated with chemotherapy and radiation therapy had significantly more serous or clear cell histology and advanced stage of disease compared to patients who were treated with radiotherapy only (5). In our patients, who more commonly had endometrioid and stage IIIA disease, OS improved from 11% without any treatment to 65.2% with sequential chemotherapy and radiation therapy (p<0.00005), suggesting that there may be survival benefit with sequential chemotherapy and radiation therapy.

Our data may reflect the benefit of adjuvant radiation therapy in preventing local relapse, particularly when combined with sequential chemotherapy. This contrasts with the GOG-258 trial where there was no significant difference in overall survival or relapse-free survival with chemoradiation compared to chemotherapy alone (12). However, the study found that less frequent locoregional relapses were found in patients who received both chemotherapy and radiation therapy compared to patients who received chemotherapy alone. Patients receiving chemoradiotherapy in the GOG-258 trial received 2 cycles of cisplastin followed by 4 cycles of carboplatin/paclitaxel, with only 75% of patients completing the 4 cycles. Patients in the chemotherapy only regimen received 6 cycles of carboplatin/paclitaxel, consistent with the standard of care. When analyzing relapse in patients who received 6 cycles of carboplatin/paclitaxel in addition to adjuvant radiation therapy, our study supports a decrease in relapse with a 38% relapse rate in patients who received adjuvant radiation therapy compared to 63% in patients who did not receive adjuvant radiation therapy. Given acceptable tolerability of pelvic radiation reported in prospective trials, our findings support continued use of sequential radiation therapy with chemotherapy.

Molecular classification is important in determining treatment (3). For example, patients with POL-E or d-MMR mutations do not seem to benefit from adjuvant therapy, in particular stage I and II (3). However, given level I evidence that adjuvant therapy adds survival benefit to stage III, and with a lack of well-powered robust evidence to support omission of adjuvant therapy in all molecular subtypes of stage III, clinicopathologic characteristics still play a role in determining risk. Our data highlights that age and stage III endometrial cancer sub-stages may not have a significant impact on survival (HR 1.03 and 0.72-1.11, respectively). This observation suggests that carefully selected patients, regardless of age or sub-stage, may benefit from adjuvant chemotherapy and radiation therapy. Although previous studies have observed progression free survival to decrease in patients who were 60+ years old, the data are confounded by the lack of standardization in the adjuvant treatment modalities within the different institutions in the studies (12). Younger, healthier patients may be more likely to be offered and receive both adjuvant chemotherapy and radiation therapy and have improved survival (12). With adherence to standardized BC guidelines (13), our results suggest that stage III endometrial cancer is high risk, regardless of histology subtype, and the role of adjuvant chemotherapy and radiation therapy should be discussed carefully for each subtype of patients.

Our study should be interpreted in the context of limitations of the study design. With a retrospective study design, it is not possible to control for confounders. Our study aimed to minimize the effect of confounding variables by including all consecutive patients and reviewing multivariate analysis. The number of patients included in our study may be limited for extensive analysis, even though we collected population data from multiple centers within British Columbia. Heterogeneity of cancer histology subtypes may limit interpretation in our study. Histological subtype could influence response to adjuvant therapy and was not assessed independently. Lastly, molecular classification was not used to stratify risk in our cohort.

In FIGO stage III endometrial cancer patients, use of both chemotherapy and radiation therapy is associated with improved DFS and OS and therefore should be recommended in all eligible patients after resection. Our study suggests that eligible patients may have improved outcomes with sequential adjuvant chemotherapy and radiation therapy, regardless of age or stage III endometrial cancer sub-stage, despite a lack of level 1 evidence to demonstrate this to date. Future studies should evaluate response to adjuvant therapy based on histological subtypes.

## Data availability statement

The original contributions presented in the study are included in the article/**Supplementary Material**. Further inquiries can be directed to the corresponding author.

## Ethics statement

The studies involving human participants were reviewed and approved by BC Cancer Agency Research Ethics Board. Written informed consent for participation was not required for this study in accordance with the national legislation and the institutional requirements.

## Author contributions

NT - Manuscript writing. AK - Conception and design, administrative support, and manuscript writing. GG - Conception and design, administrative support, and manuscript writing. AT - Conception and design, administrative support, and manuscript writing. CH - Conception and design, administrative support, and manuscript writing. JK - Conception and design, administrative support, and manuscript writing. All authors contributed to the article and approved the submitted version.
