# Supplementary material for: Impact of the adjuvant management and risk factors on survival in FIGO stage 3 endometrial cancer patients
Source: Front Oncol. 2023 Apr 6;13:1035511. doi: 10.3389/fonc.2023.1035511 (PMC10117830; doi:10.3389/fonc.2023.1035511)
Supplement: Supplementary file 1 [file DataSheet_1.docx]

Supplementary Material

## Supplementary Figures

stylefix**
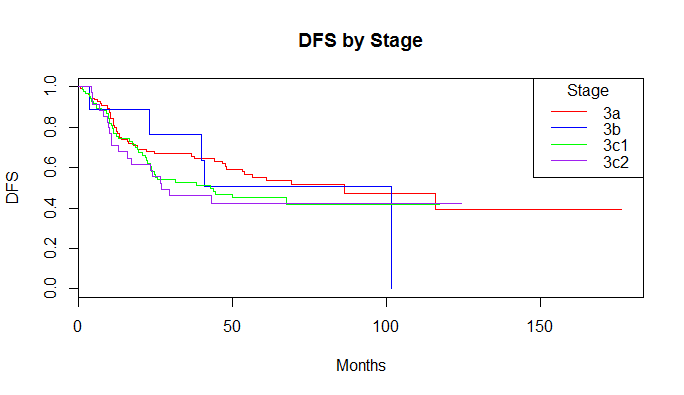
**

**Figure 1:** Disease-free survival by stage (p = 0.966)
5-year DFS: stage 3A = 46.7% (38.1.8-57.4.3); 3B = 50.8% (25.7-100); 3C1 = 44% (33.9-56.4); 3C2 = 42% (27.8-63.5)
10-year DFS: stage 3A = 27.3% (16.5.8-45.1); 3B = N/A; 3C1 = N/A; 3C2 = 42% (27.8-63.5)


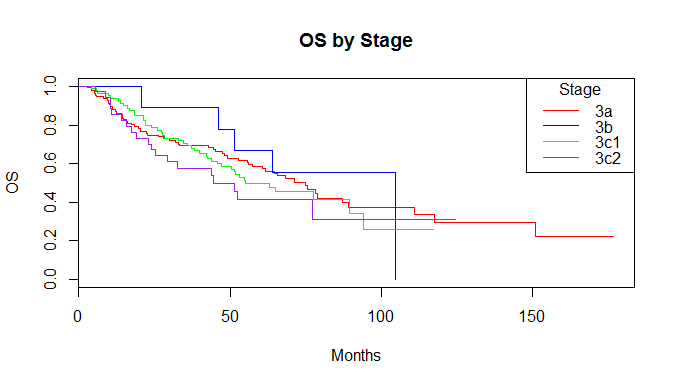


Figure 2: Overall survival by stage (p = 0.672)
5-year OS: stage 3A = 58.5% (49.8-68.7); 3B = 66.7% (42-100); 3C1 = 49.9% (39.7- 62.7); 3C2 = 41.6% (26.9-64.3)
10-year OS: stage 3A = 29.7% (19-46.4); 3B = N/A; 3C1 = N/A; 3C2 = 31.2% (15.3-63.7)

## Supplementary Tables

Table 1: Patient and disease characteristics.

| **Characteristic** | **Type** | **Number of patients** | **Percentage** |
| --- | --- | --- | --- |
| Stage | 3A | 132 | 50.6% |
|  | 3B | 9 | 3.4% |
|  | 3C1 | 85 | 32.6% |
|  | 3C2 | 35 | 13.4% |
| FIGO grade | 1 | 39 | 14.9% |
|  | 2 | 73 | 28.0% |
|  | 3 | 147 | 56.3% |
|  | Unknown | 2 | 0.8% |
| Histology | Endometrioid | 160 | 61.3% |
|  | Serous carcinoma | 35 | 13.4% |
|  | Clear cell | 10 | 3.8% |
|  | Mucinous | 1 | 0.4% |
|  | Undifferentiated/carcinosarcoma/MMMT | 5 | 1.9% |
|  | Mixed | 48 | 18.4% |
|  | Unknown | 2 | 0.8% |
| Myometrial invasion | >50% | 170 | 65.1% |
| Lymphovascular invasion | Present | 162 | 62.0% |

Table 2: Multivariate analysis of age, stage, grade, myometrial invasion, and adjuvant radiotherapy or chemotherapy on DFS and OS.

|  | DFS | | OS | |
| --- | --- | --- | --- | --- |
|  | HR | p-value | HR | p-value |
| Age | 1.03(1.01-1.05) | 0.001 | 1.03(1.01-1.05) | 0.01 |
| Stage (vs. 3a)  3b  3c1  3c2 | 1.37(0.42-4.46)  0.78(0.51-1.19)  0.85(0.49-1.49) | 0.60  0.25  0.57 | 1.11(0.33-3.68)  0.72(0.46-1.12)  1.09(0.61-1.94) | 0.87  0.15  0.78 |
| Grade (vs. Grade 1)  Grade 2  Grade 3 | 1.42(0.65-3.1)  4.77(2.37-9.63) | 0.38  <0.0001 | 1.53(0.66-3.55)  6.28(2.87-13.75) | 0.33  <0.00001 |
| MyoInvas | 2.14(1.37-3.34) | 0.0008 | 2.47(1.52-4) | 0.0003 |
| XRT | 0.45(0.29-0.71) | 0.0005 | 0.44(0.27-0.73) | 0.001 |
| Chemo | 0.44(0.29-0.66) | 0.0001 | 0.57(0.36-0.91) | 0.02 |

Table 3: Pattern of relapse.

| **Adjuvant RT** | Number of patients | In field vaginal/pelvic relapse | Out of field vaginal/pelvic relapse | Abdominal/pelvic relapse (in and out of fields) | Distant sites or with visceral metastases | Abdominal/pelvic relapse (surgical site with no RT) | More than 1 |
| --- | --- | --- | --- | --- | --- | --- | --- |
| Yes | 209 | 11 (5%) | 5 (2%) | 23 (11%) | 23 (11%) | N/A | 18 (9%) |
| No | 75 | N/A* | N/A | N/A | 9 (12%) | 29 (39%) | 9 (12%) |

N/A*: not applicable
